# Practicing quality nephropathology in a developing country; challenges and solutions

**DOI:** 10.12860/jnp.2015.12

**Published:** 2015-07-01

**Authors:** Muhammed Mubarak

**Affiliations:** ^1^Histopathology Department, Sindh Institute of Urology and Transplantation, Karachi, Pakistan

**Keywords:** Nephropathology, Renal biopsy, Autopsy, Morphology, Developing country

**Implication for health policy/practice/research/medical education:**

Nephropathology is a highly specialized field of surgical pathology that deals with the diagnosis, management and prognostication of medical renal diseases. It is important for the nephropathologists to have a close liaison with the nephrologists, immunologists, molecular biologists, and more recently geneticists in order to facilitate accurate diagnosis of the renal diseases.

Nephropathology is a highly specialized field of surgical pathology that deals with the diagnosis, management and prognostication of medical renal diseases (1). It is considered the most difficult area of pathology by most surgical pathologists, not only in developing part of the world but also the developed world. There are several reasons for this thinking. Nephropathological diagnosis is nowadays almost always made on percutaneous needle biopsies, a tissue sample often abhorred by the majority of diagnostic pathologists, but one which has enriched the study of the natural course and the evolution of the nephrological diseases (2-4). Prior to its widespread use, the study of renal pathology was based almost entirely on autopsy pathology, which in most instances, showed only extreme examples of pathological processes in acute renal failure or end-stage renal disease (ESRD) with chronic renal failure, with little insight of the preceding events leading to the final picture (2). With percutaneous renal biopsies it became possible to study the disease process at different stages of the disease and often during the early phase. In some centers, serial biopsies in individual patients illustrated the progression of the disease process very nicely. In addition, as will be discussed shortly hereafter, the renal disease proved an ideal topic for the application of two techniques which were also relatively new at the time; electron microscopy (EM) and the use of antibodies to localize antigens in tissue sections (2). With this came the challenges of designating the disease processes that were hitherto undiscovered by the autopsy-based practice and labeling the histological lesions not previously reported in the autopsy-based literature. Moreover, a strong need was felt for the standardization of the nomenclature of the renal pathological lesions and uniform system for classification of the diseases to facilitate international communication and comparison of studies. These challenged were tackled amicably and admirably by the World Health Organization (WHO) Collaborating Center for the Histological Classification of Renal Diseases, established in 1974 and by some individual pathologists such as Robert H. Heptinstall (5,6).

The renal biopsy tissue also proved an ideal substrate for the application of two ancillary techniques of immunohistochemistry (IHC) and EM. These techniques proved immensely helpful in elucidating the pathogenetic and minute pathological details of the glomerular diseases (2,5,6). Soon they became part of the routine tools for the investigation of glomerular diseases in most laboratories in the developed world (2,4). However, these investigations also entailed correlation of the data obtained from these investigations with the morphology and the clinical features of the disease to reach a complete diagnosis. This additional work-up of the renal biopsy tissue and the requirement for separate specimens in specialized fixatives for these investigations put extra burden on the nephropathologists.

In addition, the nephropathological diagnosis often evolves from a morphological diagnosis to disease diagnosis to etiological diagnosis, which constitutes the key to treatment. It can change from, for example, minor glomerular abnormalities to minimal change disease on negative immunofluorescence (IF) and fusion of foot processes on EM. Alternatively, the same morphological lesion may change to thin basement membrane disease if EM demonstrates uniform thinning of this structure in a child with microscopic hematuria (6). The burden of etiologic diagnosis often lies with the nephrologists, rather than nephropathologists and requires additional clinical, laboratory and imaging investigations. It is important for the nephropathologists to have a close liaison with the nephrologists, immunologists, molecular biologists, and more recently geneticists in order to facilitate accurate diagnosis of the renal diseases (7,8). Only in very few instances, final etiological diagnosis can be made on the biopsy tissue.

Thus, in effect, renal pathology diagnosis is a correlative diagnosis. It cannot be based on just one modality of investigations. Morphology which forms the mainstay of diagnostic pathology in many other systems of the body cannot form the sole basis of diagnosis in renal diseases. It represents the glomerular response to various forms of injury and is not equivalent to specific disease diagnosis. It forms the starting point for further pathological evaluation of renal biopsy material. Information from all modalities of diagnosis need to be integrated, correlated and synthesized to reach the best possible diagnosis (2,9-13).

But the quality of the information gained from a renal biopsy study depends on numerous factors, including the size and type of renal biopsy sample, its fixation, processing, microtomy, and staining. The thickness of sections, quality of the reagents, and the expertise of the technical staff are of utmost importance for proper evaluation of the morphological lesions. No compromise should be made on the quality of fixative, quality of processing reagents, thin sectioning of the paraffin embedded tissue and the quality of staining reagents, if proper nephropathology is to be practiced. These issues pose significant problems in developing countries. Lack of funds, lack of training programs for technicians and problems of procurement of analysis-grade reagents are recurring problems in most developing countries (1,2).

There are dedicated nephropathology laboratories in most nephrological centers in developed world. These are equipped with all the necessary diagnostic armamentarium for the complete pathological evaluation of renal biopsies. On the other hand, nephropathology receives little attention in most of the surgical pathology laboratories in the developing nations. These laboratories are forced to report renal biopsies with light microscopic examination alone. The problem is often exacerbated by the thicker sections due to suboptimal processing in poor quality chemicals. Just a few examples suffice to illustrate this point; alcohol is a particular problem because it is a banned item in most developing countries. A good quality silver stain is almost impossible to achieve with cheap laboratory reagents. Moreover, the subspecialty of nephrology as a separate specialty of medicine has lagged behind in establishing its foothold in developing countries, and since nephropathology caters to the needs of this subspecialty, it too has been slow to foster in these regions of the world. There are usually no external quality assurance programs in developing countries to oversee the control of these laboratories. But, now more and more nephropathology laboratories are doing quality work at par with the international standard in the developing nations also (1). It is interesting to note the developing countries have outpaced their developed counterparts in publishing a biomedical journal dedicated to the field of nephropathology. *Journal of Nephropathology* was launched in 2012 and in a brief period of time has succeeded in obtaining indexation with PubMed Central (PMC) and Scopus (14).

One cost-effective and most efficient solution to the above challenges lies in establishing a few centers of excellence for nephropathology in the country with all the necessary diagnostic facilities and these should serve as referral centers catering to the regional needs. Small laboratories can and should try to preserve tissue for IF and EM and establish a working relationship with the referral centers to which they can refer the tissue and whose methods they can adopt for the initial processing. This will be of considerable help in optimal practice of nephropathology in a developing country.

In summary, there are numerous challenges in practicing quality nephropathology in developing countries in general diagnostic surgical pathology laboratories. This can be overcome by establishing one or a few specialty-specific centers of excellence where cases can be referred from all over the country and establishing a working relationship with non-specialist laboratories.

## Author’s contribution

MM was the single author of this manuscript.

## Conflicts of Interest

The author declared no competing interests.

## Funding/Support

None.
